# Clinical outcome, risk assessment, and seasonal variation in hospitalized COVID-19 patients—Results from the CORONA Germany study

**DOI:** 10.1371/journal.pone.0252867

**Published:** 2021-06-17

**Authors:** Nele Gessler, Melanie A. Gunawardene, Peter Wohlmuth, Dirk Arnold, Juergen Behr, Christian Gloeckner, Klaus Herrlinger, Thomas Hoelting, Ulrich-Frank Pape, Ruediger Schreiber, Axel Stang, Claas Wesseler, Stephan Willems, Charlotte Arms, Christoph U. Herborn

**Affiliations:** 1 Department of Cardiology and Internal Intensive Care Medicine, Asklepios Hospital St. Georg, Hamburg, Germany; 2 Asklepios Proresearch, Research Institute, Hamburg, Germany; 3 Semmelweis University, Budapest, Hungary; 4 Department of Hematology, Oncology, Palliative Care Medicine and Rheumatology, Asklepios Hospital Altona, Hamburg, Germany; 5 Department of Pneumology, Member of the German Center for Lung Research, Asklepios Hospital Munich-Gauting, Gauting, Germany; 6 Department of Internal Medicine, Asklepios Hospital Oberviechtach, Oberviechtach, Germany; 7 Department of Internal Medicine—Gastroenterology, Asklepios Hospital Nord-Heidberg, Hamburg, Germany; 8 Department of Internal Medicine—Cardiology and Pneumology, Asklepios Hospital Wandsbek, Hamburg, Germany; 9 Department of Internal Medicine—Gastroenterology, Asklepios Hospital St. Georg, Hamburg, Germany; 10 Department Anesthesiology and Intensive Care Medicine, Asklepios Hospital West, Hamburg, Germany; 11 Department of Oncology and Palliative Care Medicine, Asklepios Hospital Barmbek, Hamburg, Germany; 12 Department of Pneumology, Asklepios Hospital Harburg, Hamburg, Germany; 13 Asklepios Hospitals GmbH & Co. KGaA, Hamburg, Germany; University Magna Graecia of Catanzaro, ITALY

## Abstract

**Background:**

After one year of the pandemic and hints of seasonal patterns, temporal variations of in-hospital mortality in COVID-19 are widely unknown. Additionally, heterogeneous data regarding clinical indicators predicting disease severity has been published. However, there is a need for a risk stratification model integrating the effects on disease severity and mortality to support clinical decision-making.

**Methods:**

We conducted a multicenter, observational, prospective, epidemiological cohort study at 45 hospitals in Germany. Until 1 January 2021, all hospitalized SARS CoV-2 positive patients were included. A comprehensive data set was collected in a cohort of seven hospitals. The primary objective was disease severity and prediction of mild, severe, and fatal cases. Ancillary analyses included a temporal analysis of all hospitalized COVID-19 patients for the entire year 2020.

**Findings:**

A total of 4704 COVID-19 patients were hospitalized with a mortality rate of 19% (890/4704). Rates of mortality, need for ventilation, pneumonia, and respiratory insufficiency showed temporal variations, whereas age had a strong influence on the course of mortality. In cohort conducting analyses, prognostic factors for fatal/severe disease were: age (odds ratio (OR) 1.704, CI:[1.221–2.377]), respiratory rate (OR 1.688, CI:[1.222–2.333]), lactate dehydrogenase (LDH) (OR 1.312, CI:[1.015–1.695]), C-reactive protein (CRP) (OR 2.132, CI:[1.533–2.965]), and creatinine values (OR 2.573, CI:[1.593–4.154].

**Conclusions:**

Age, respiratory rate, LDH, CRP, and creatinine at baseline are associated with all cause death, and need for ventilation/ICU treatment in a nationwide series of COVID 19 hospitalized patients. Especially age plays an important prognostic role. In-hospital mortality showed temporal variation during the year 2020, influenced by age.

**Trial registration number:**

NCT04659187.

## Introduction

The outbreak of the severe acute respiratory syndrome coronavirus 2 (SARS-CoV-2) causing novel coronavirus disease 2019 (COVID-19) infected millions of people in a worldwide pandemic situation after it was first discovered in December 2019 [1, 2].

In Germany, the first case was observed on 27 January 2020. Infection numbers increased with a peak at the end of March (first wave or phase 1), low numbers during summer (phase 2) and an increase in October with a maximum in December 2020 (second wave or phase 3). The first SARS-CoV-2 variant of concern was detected at the end of December 2020 in Germany [3]. A governmental lockdown was implemented at 23 March until 20 April 2020 with closing of schools, restaurants, most shops, etc. Several restrictions remained until 11 Mai 2020. SARS-CoV-2 testing was recommended for hospitals and retirement homes on 15 October and new restrictions were implemented on 2 November with a new lockdown on 16 December 2020. Despite high numbers of hospitalized patients with a maximum of 5.643 intensive care patients with COVID-19 in December, hospitals were able to accept all patients and provide the same level of care throughout the pandemic [4].

After approximately one year of pandemic, it is possible to evaluate first observations regarding temporal changes of SARS-CoV-2 infection rates, which were reported previously [5]. Dennis et al. documented a decrease in mortality in a large cohort of intensive care patients from 1 March 2020 until 27 June 2020, assumed to be caused by introduction of effective treatments, improved physician understanding of the disease process, and a falling critical care burden [6]. However, temporal variations of in-hospital mortality in COVID-19 over a longer period of time are widely unreported.

While most SARS-CoV-2 infected patients have non-severe symptoms, 14% develop dyspnea and hypoxemia, which can lead to severe or fatal courses [7]. The often-observed silent hypoxemia and rapid worsening of symptoms, with patients becoming seriously ill in a short period of time, requires the need to detect patients at risk fast and easily. Previous studies described older age, male sex, cardiovascular disease, hypertension, and diabetes as possible risk factors [8–10]. Nevertheless, this crisis has led to the publication of multiple, heterogeneous results regarding the impact of clinical indicators on disease severity in hospitalized COVID-19 patients with different singular or combined endpoints. Structured, integrated approaches for data analysis are sparse.

The aim of this multicenter cohort study was to assess the impact of possible baseline risk factors on disease progression and on mortality with an integrated approach for data analysis. Additionally, we present temporal data for the entire year 2020 regarding in-hospital mortality, morbidity, and need for mechanical ventilation.

## Methods

### Study design

The “CORONA Germany”—*Clinical Outcome and Risk in hospitalized COVID-19 patients—*study (ClinicalTrials.gov, NCT04659187) is a multicenter, observational, prospective, epidemiological cohort study. It was conducted in 45 hospitals across Germany that were all part of the same hospital network (see S1 Appendix for list of hospitals). The trial was without funding and investigator-initiated; the steering committee was responsible for design, execution, and conduct of the study. The statistical analyses and interpretation of the data was approved by all members of the steering committee. An endpoint committee, provided by the networks research institute, reviewed all study endpoints. All data were matched to and validated by the networks’ quality management data base. The authors attest to the accuracy of the data and of all analyses. The ethics committee of the General Medical Council (Aerztekammer) for the city of Hamburg and the ethics committee of the General Medical Council (Aerztekammer) for the city of Munich approved the study and determined that this work was exempt from human subjects’ research regulations since all information was collected on a fully anonymized basis. Therefore, the ethics committees waived the need for participant consent.

### Study participants and patients

Enrollment of all consecutive hospitalized patients testing positive for SARS CoV-2 (PCR, polymerase chain reaction test) was the key inclusion criterion. Patients with negative SARS CoV-2 testing and outpatients were excluded from this study.

The total sample size was open, reflecting the development of the pandemic situation, missing historic data and the explorative design of the study.

This dataset included all patients coded with international statistical classification of diseases and related health (ICD) for coronavirus SARS CoV-2 infection and need for hospitalization. Data about age, gender, outcome, necessity for mechanical ventilation (invasive and non-invasive ventilation), and all ICD-coded main (co-)diagnoses were given for the total study cohort. Patients were enrolled from the start of pandemic in February 2020 until 1 January 2021, while only patients with a complete hospital stay (until discharge or death) were analyzed.

Additionally, the predefined cohort from Hamburg and Gauting, including seven of the participating centers, provided a second data set consisting of more detailed information collected from 1 March until 15 September 2020. These seven centers are specialized hospitals regarding intensive care, cardiovascular care, and research. They all provided access to electronic patient charts of the same hospital information technology network. Therefore, development of a comprehensive second data set was feasible. Data entry was performed in a structured way, anonymously. The data fields were pre-formatted and periodic data checks ensured a high data quality. This dataset included n = 45 variables regarding baseline symptoms, baseline vital parameters, baseline laboratory findings, prior medication, and preexisting comorbidities. Each study participant was followed up for the whole length of the hospital stay.

### Endpoints

The aim of this study was to assess the impact of possible risk factors on disease progression and on mortality. Therefore, the primary endpoint was assessment of disease severity defined by three stages:

1. non-survivors (fatal disease),
2. requirement of mechanical ventilation and/or ICU admission and afterwards successful hospital discharge (severe disease), and
3. recovery after a hospital stay solely on a normal, non-ICU ward (mild disease).

For simplification, these will be referred to as fatal, severe, and mild disease in the following manuscript. The impact of risk factors on COVID-19 disease in hospitalized patients was assessed in two steps. First, effects on fatal and severe disease were examined. Second, predictors on overall survival for patients with severe but non-fatal disease (ventilation and/or ICU) were analyzed.

Only baseline data i.e. variables regarding baseline symptoms, baseline vital parameters, baseline laboratory findings, prior medication, and preexisting comorbidities were investigated as possible risk factors. Potential predictive variables were: age, body mass index, fever at admission (defined cut-off 38·5°C), presence of dyspnea at admission, presence of cough at admission, baseline vital parameters (including respiratory rate, oxygen saturation by pulse oximetry (SpO2), systolic and diastolic blood pressure), laboratory findings at admission (leukocyte count, lymphocyte count, d-dimer level, creatinine, C-reactive protein, procalcitonin, activated partial thromboplastin time, potassium level, initial lactate dehydrogenase), coexisting comorbidities (presence of cardiomyopathy, hypertension, diabetes mellitus, vascular disease, dyslipidemia, pulmonary disease, chronic kidney disease, active tumor disease), prior medication (ACE-inhibitor, angiotensin receptor blocker (ARB), statins, antiplatelet therapy, oral anticoagulation, diuretics, proton pump inhibitors, chemo-/immunosuppression and/or targeted therapy).

After statistical analyses, the detected risk factors were depicted in a nomogram (risk score), which is a graphical tool for manually obtaining predictions from the fitted model. Point scores can be obtained for each predictor and the user should add the points manually before reading predicted values on the final axis of the nomogram. With this tool it is possible to calculate the individual risk score for each patient.

Ancillary endpoints were temporal trends for mortality, need for mechanical ventilation (invasive and non-invasive), and morbidity (respiratory insufficiency and pneumonia, as defined by current guidelines [11]) over the observational period. Additional analyses were rates, baseline characteristics, and outcomes of patients receiving palliative care.

Palliative care was defined according to the World Health Organization (WHO) as an approach that improves the quality of life of patients and their families facing the problems associated with life-threatening illness, through the prevention and relief of suffering by means of early identification and impeccable assessment and treatment of pain and other problems, physical, psychosocial, and spiritual [12].

### Statistical analysis

#### Descriptives

Demographic data, symptoms, vital data, laboratory data, cardiac marker, blood gas analysis, comorbidities, and medication, documented at baseline, were summarized descriptively. Continuous data are shown as means +/- standard deviations and medians [25th and 75th percentiles]. Categorical data are presented as proportions and frequencies (data presented in Tables 1–3).

**Table 1:** Patient characteristics complete study cohort (N = 4704).

	N	(N = 4704)
**Baseline**		
Age (years)	4696	68+/-18
Gender (male)	4704	0.52 (2439)
**Endpoints**		
Death (N)		0.19 (890)
Patients with need of mechanical ventilation (N)	4704	0.12 (572)
**Coexisting Diagnoses (ICD code), (N)**		
Acute Respiratory Distress Syndrome (J80)		0.06 (271)
Respiratory insufficiency (J96·0, J96·1)		0.47 (2228)
Pneumonia (J10—J18)		0.56 (2629)
Acute kidney failure (N17)		0.13 (625)
Liver failure (K72)		0.01 (55)
Sepsis (A40, A41, R65, A39·2, A39·4, B37·7)		0.08 (371)
**Procedural data**		
Duration of mechanical ventilation (hours, median)	572	63/194/414
Duration of hospitalization (days)	2132	3/ 7/14
Kidney replacement therapy (8–853, 8–854, 8–855, 8–857, 8-85a)		0.04 (174)

N are number of non-missing values. Continuous data are shown as mean ± standard deviation, first quartile/median/third quartile. Categorical data as proportions (n).

**Table 2:** Patient characteristics for patients receiving palliative care (palliative patients) and patients not receiving palliative care (non-palliative patients) (N = 4704).

	N	Palliative patients (N = 423)	Non-palliative patients (N = 4281)
**Baseline**			
Age (years)	4696	82.7+/- 8.7	66.8+/-18.4
Gender (male)	4704	0.45 (192)	0.52 (2247)
**Endpoints**			
Death (N)		0.75 (316)	0.13 (574)
Patients with need of mechanical ventilation (N)	4704	0.16 (67)	0.12 (505)
**Coexisting Diagnoses (ICD code), (N)**			
Acute Respiratory Distress Syndrome (J80)		0.07 (31)	0.06 (240)
Respiratory insufficiency (J96·0, J96·1)		0.71 (299)	0.45 (1929)
Pneumonia (J10—J18)		0.75 (318)	0.54 (2311)
Acute kidney failure (N17)		0.30 (128)	0.12 (497)
Liver failure (K72)		0.03 (12)	0.01 (43)
Sepsis (A40, A41, R65, A39·2, A39·4, B37·7)		0.14 (60)	0.07 (311)
**Procedural data**			
Duration of mechanical ventilation (hours, median)	572	58/150/312	68/205/420
Duration of hospitalization (days)	2132	4/ 8/15	3/ 7/14
Kidney replacement therapy (8–853, 8–854, 8–855, 8–857, 8-85a)		0.05 (21)	0.04 (153)

N are number of non-missing values. Continuous data are shown as mean ± standard deviation, first quartile/median/third quartile. Categorical data as proportions (n).

**Table 3:** In-hospital mortality for each calendar month 2020, complete study cohort (n = 4704).

2020	(n = 4704)
February	0.8 (4/5)
March	0.136 (53/389)
April	0.224 (125/558)
May	0.138 (17/123)
June	0.03 (1/33)
July	0.043 (2/46)
August	0.04 (3/75)
September	0.167 (21/126)
October	0.161 (110/684)
November	0.19 (285/1499)
December	0.231 (269/1166)

Categorical data are shown as proportions (deaths/COVID cases).

#### Model development

The primary endpoint was analyzed in an ordinal fashion, recording the worst of the following three states: death for non-survivors, severe cases as the need for ventilation or ICU admission for survivors, or mild state as discharge from a non-ICU ward.

The missing data pattern was analyzed beforehand. Variables with more than 1/3 missing values and systematic data gaps were not taken into account for model building. Further data gaps were filled by multiple imputation using chained equations (MICE algorithm). First, an ordinal logistic regression (continuation ratio) model was applied to associate baseline data with the occurrence of disease severity. Nonlinearities in continuous variables were considered by restricted cubic splines transformations. Three knots at the 10^th^, 50^th^ and 90^th^ percentile were set. The regression model was fitted without deletion of further predictors using a penalized estimation approach.

As a second step, two models were derived 1) for the prediction of disease severity and non-surviving and 2) for the prediction of non-surviving conditional on at least severe cases, where no mild cases were taken into account. Each models was approximated from the full model to become simplified and clinically useable. The linear predictor of the logistic regression model was used as the outcome in a linear regression model. A step-down method on a least squares model was applied to simplify the model. While the full OLS model shows an R2 of 1, an approximation could theoretically be applied to any level. The final models were selected to exhibit a high R2 level and a high goodness of fit (AIC, BIC).

#### Validation of the prediction models

The logistic regression models were validated using B = 200 bootstrap samples. The training sample was used to fit the models and estimate the apparent model fit. The out-of-sample data were used to calculate the test performance of the model. The mean difference between training and test performance (overfitting in the final models) was examined using explained variation R2 and Sommers’ D rank-correlation. Validation results are shown in the S2 Appendix.

#### Model effects

The predictors of each model are shown with odds ratios and 95% confidence intervals and were summarized in forest plots. Continuous predictors are presented as interquartile-range odds ratios (upper quartile: lower quartile) and categorical predictors as simple odds ratios (current category: reference category). Partial effects in the prediction model for the cause of a disease severity and mortality are shown on a log-odds scale. Nomograms were derived from the prediction models to calculate the probability for the patients’ states. Event prediction in the nomogram was derived from a total point score that summarizes individual points (0–100) assigned from the value of each predictor.

#### Further analyses based on the entire dataset

Weekly rates of mortality, need for ventilation, pneumonia, respiratory insufficiency, and mean age are presented using smoothed local regression curves across time. In cases with missing medical history data only age was related to mortality. A one dimensional regression model was used to relate weekly changes in mean age to mortality rates.

**Fig 1:**
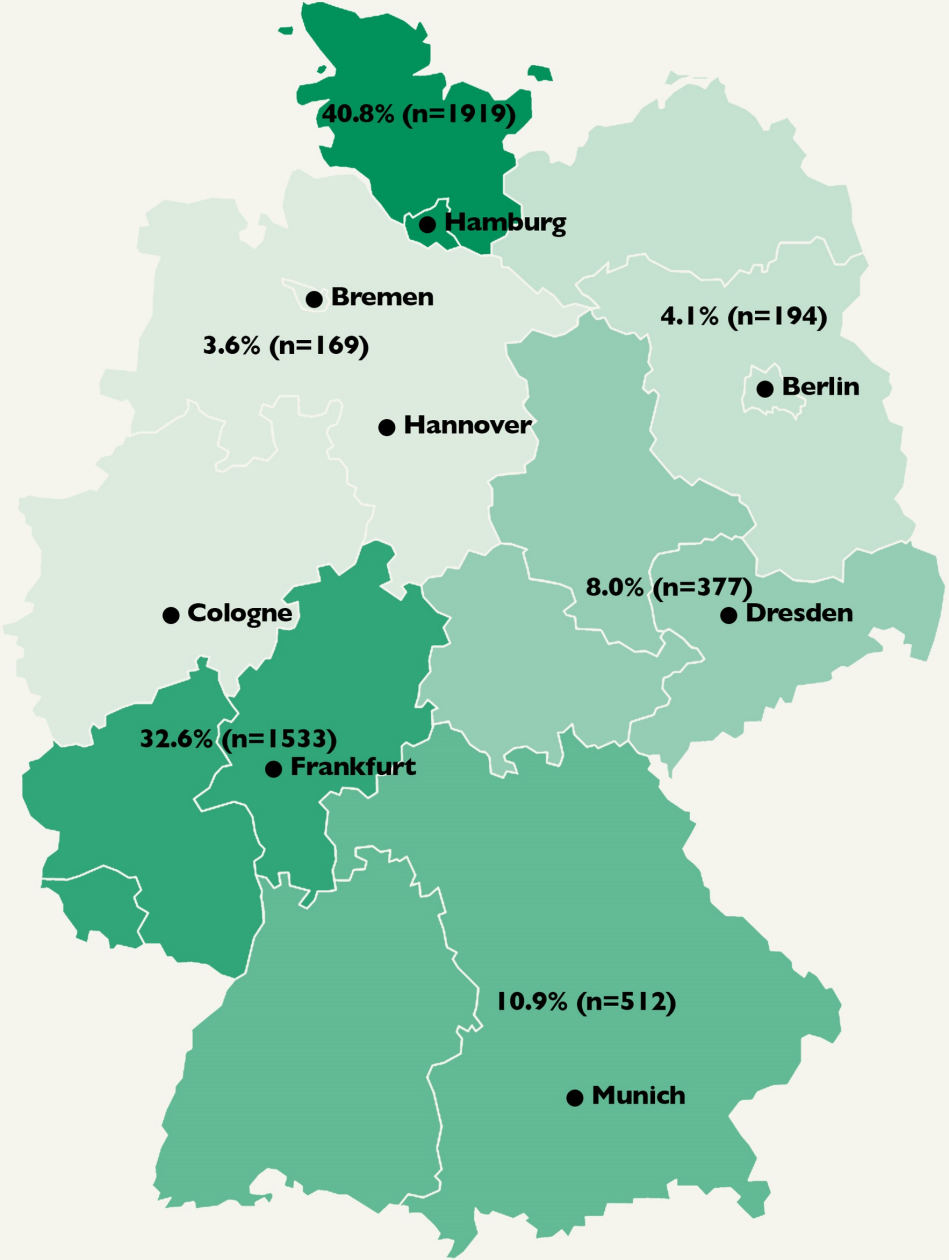
Patient distribution of the 4704 included patients in Germany. The map of Germany is divided into northern, northeastern, northwestern, south, southeastern and southwestern Germany.

**Fig 2:**
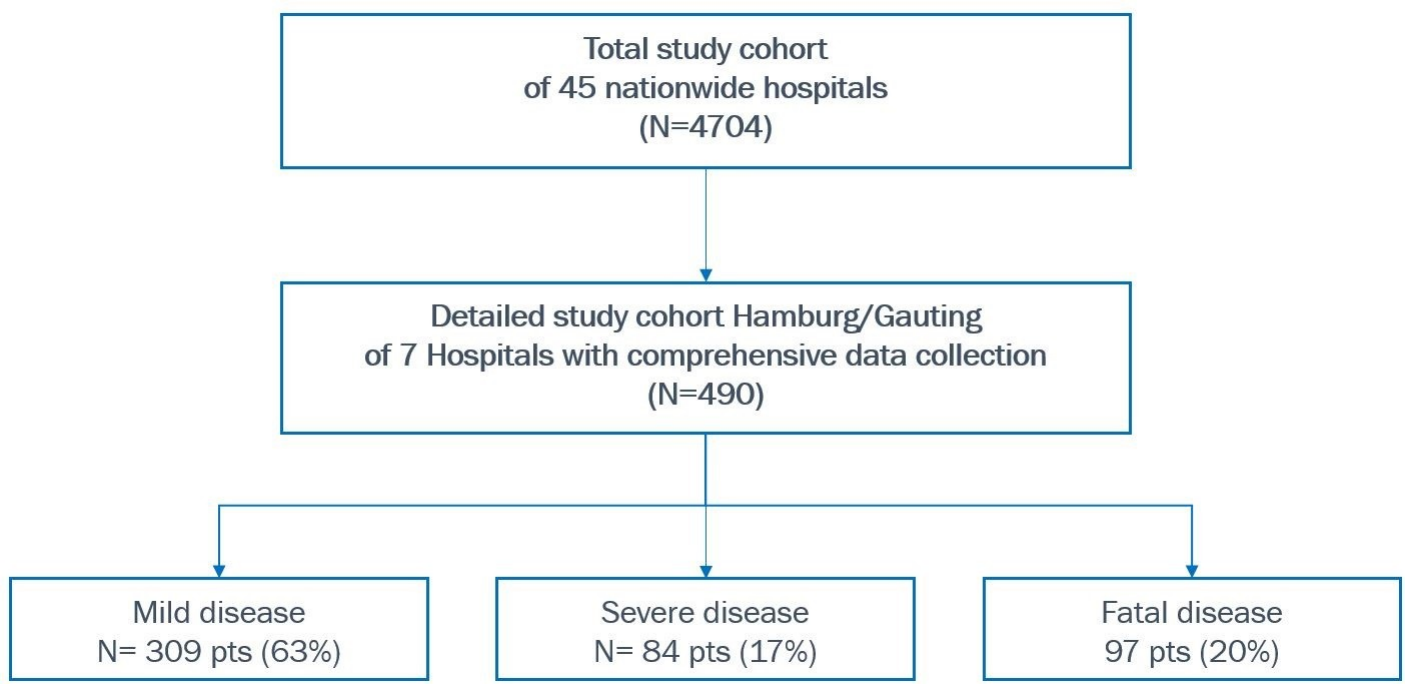
Flowchart of the trial showing the entire study population and the cohort Hamburg/Gauting with the primary endpoint mortality and severity of disease (mild, severe and fatal course).

**Fig 3:**
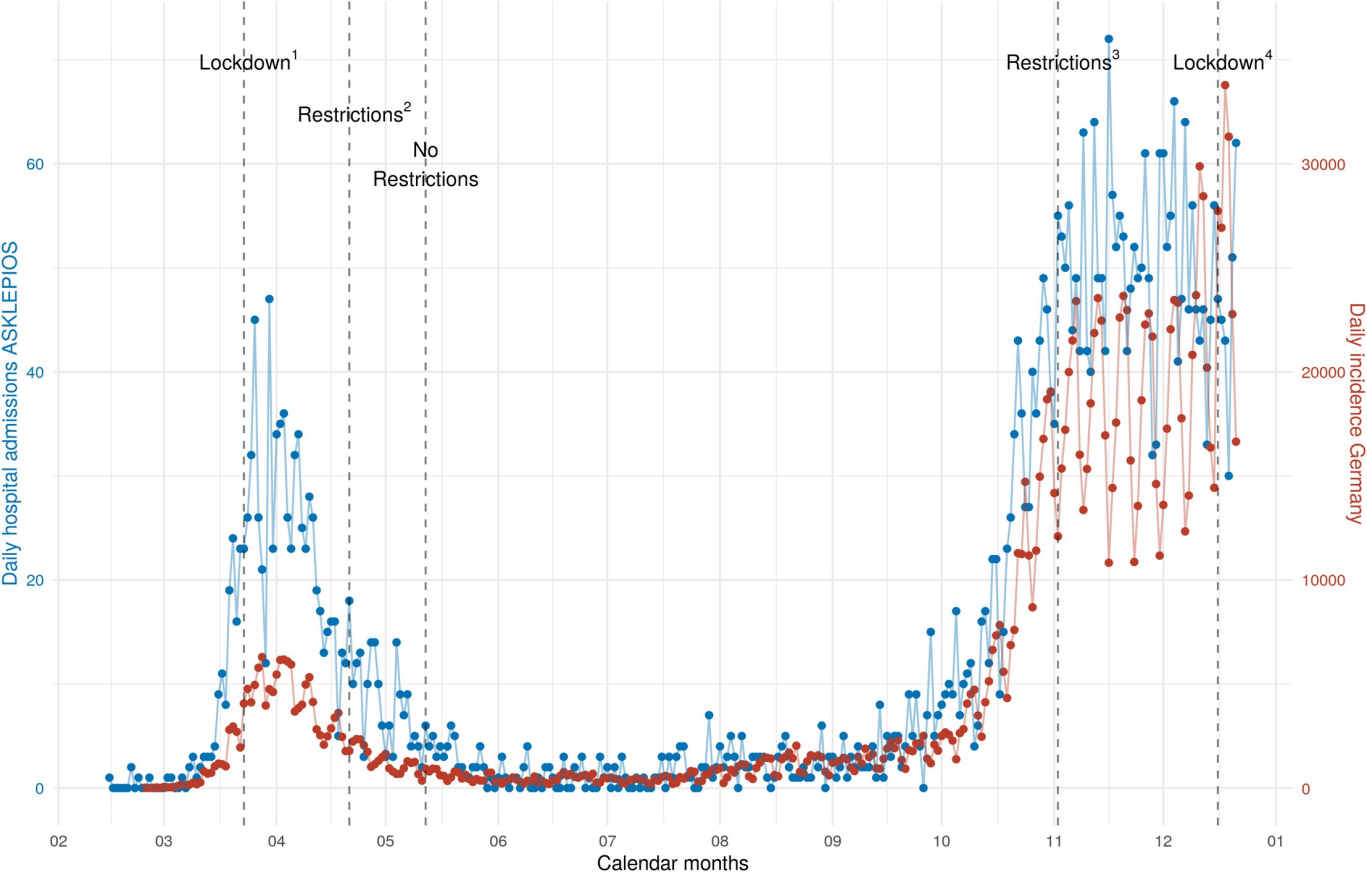
Daily admissions of COVID-19 patients to the participating 45 centers in 2020 (blue color) and daily incidence of Germany in 2020 (red color, data used with kind approval by RKI, [13]). ^1^Lockdown: Closing of restaurants, most shops, schools, kindergardens, etc.; ^2^Restrictions: First openings of shops (with restrictions), partial opening of schools, restaurants remained closed; ^3^Restrictions: Closing of restaurants, hairdressing salons, etc.; ^4^Lockdown: Additionally, closing of schools, kindergardens, several shops, etc.

**Fig 4:**
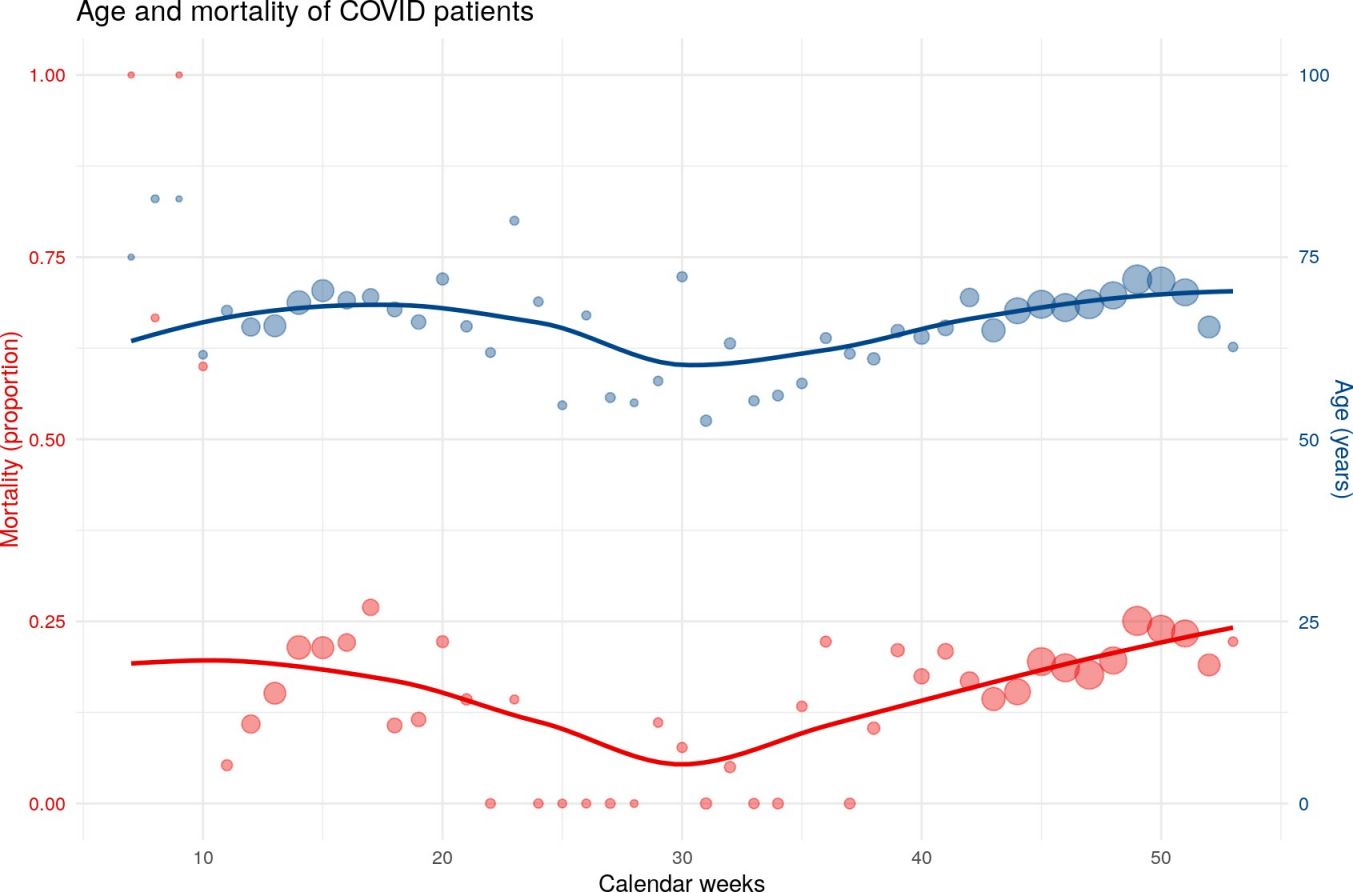
Temporal trend of mortality and age during the year 2020, based on the total study cohort (n = 4704). Dot size: small = up to 20 cases, medium = 21–40 cases, big = 41–60 cases.

**Fig 5:**
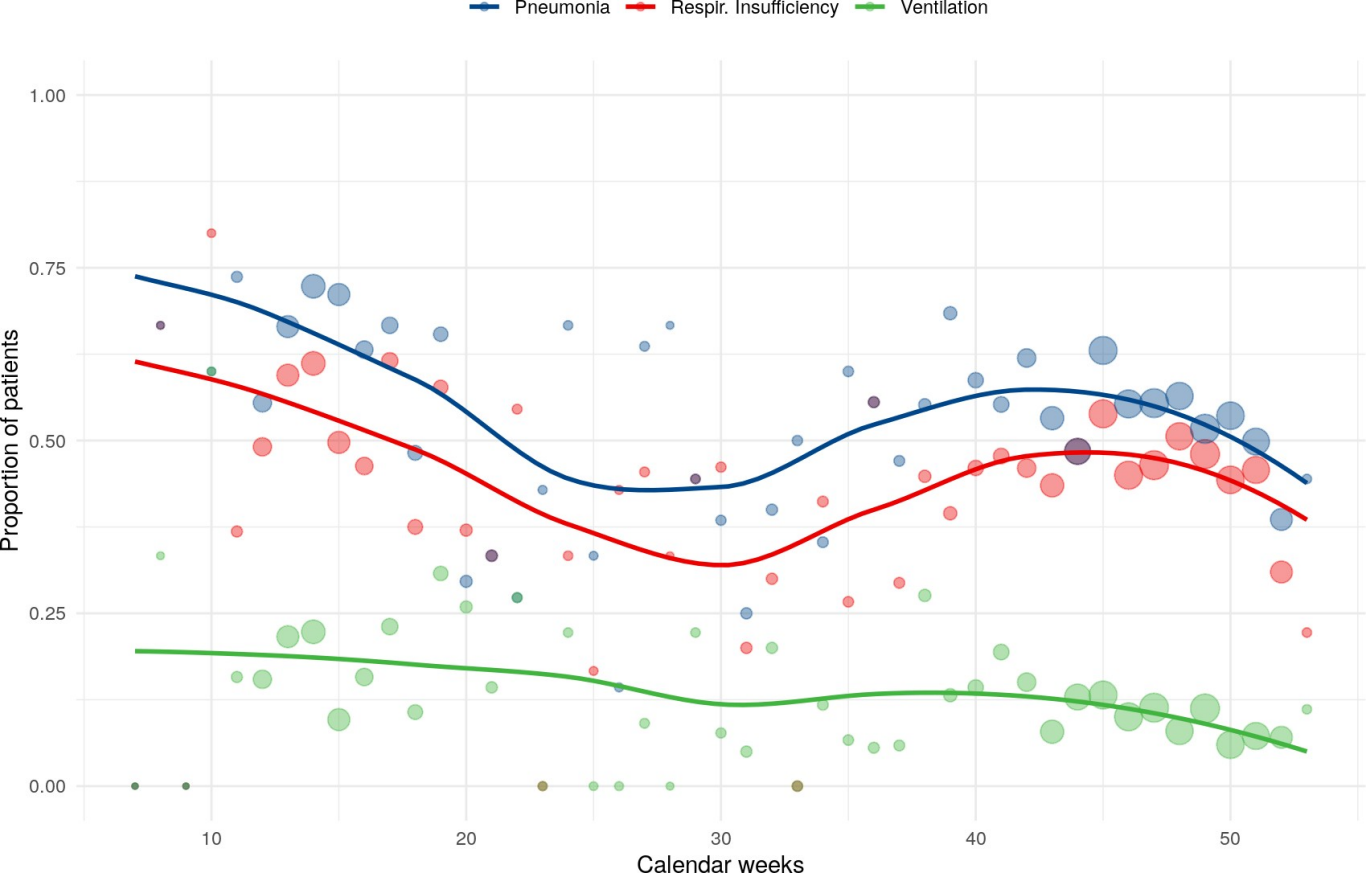
Temporal trend of pneumonia, respiratory insufficiency and need for ventilation during the year 2020, based on the total study cohort (n = 4704). Dot size: small = up to 20 cases, medium = 21–40 cases, big = 41–60 cases.

All analyses except the descriptive analyses of the whole cohort (Tables 1 and 2) and weekly rates of mortality, need for ventilation, pneumonia, respiratory insufficiency, and mean age (Table 3, Figs 1–5) were based on the Hamburg/Gauting cohort. P-values are two-sided. The significance level is 5%. All analyses were performed with R (R Core Team 2020), (see S3 Appendix for statistical literature).

## Results

### Total study cohort

A total of 4704 COVID-19 patients were included in the study (all hospitalized SARS-CoV-2 positive patients with full data set) in 45 centers (Figs 1 and 2). Table 1 shows the characteristics of the total study cohort, including age, gender, coexisting diagnoses, and procedural data. Death occurred in 890 of the 4704 patients (19%), and 572 of 4704 patients (12%) required mechanical ventilation (invasive and non-invasive ventilation) during hospitalization. Mortality in the group of patients requiring mechanical ventilation was 44.1% (252/572) compared to 15.4% (638/4132) in patients without mechanical ventilation. Of all fatal cases, 35.5% (316/890) were receiving palliative care.

Procedural data of the total study cohort showed a median mechanical ventilation time of 194 hours (interquartile range: 63–414). Kidney replacement therapy due to kidney failure was applied to 174 of 4704 patients (4%). Patients were hospitalized for a median duration of seven days (interquartile range: 3–14).

Of 4704 patients, 423 (9%) received palliative care. Table 2 shows the characteristics of this subgroup. Death occurred in 75% (316/423) and 16% (67/423) were mechanically ventilated (invasive and non-invasive ventilation) at some point during hospitalization. Palliative care patients were older with a median age of 84 years (interquartile range: 79–89) compared to non-palliative care patients (71 years [interquartile range: 55–81]). They showed higher rates of acquired comorbidities, e.g. rates of respiratory insufficiency (71% palliative vs. 45% non-palliative) and pneumonia (75% palliative vs. 54% non-palliative).

### Temporal variations

With regard to the entire study cohort of 4704 patients, Fig 3 shows the temporal variations of hospital admissions during the year 2020 with a first peak in April, low numbers during summer, and a second peak in November and December 2020.

Mortality varied during the year: rates of 22.4% in April 2020 decrease to 3% in June 2020 with an increase at the end of the year to up to 23.1% (Table 3, Fig 4). The rates of pneumonia, respiratory insufficiency, and the need for mechanical ventilation showed similar trends (Fig 5). The temporal variations of mortality rates and age were associated (p = 0.029).

### Hamburg/Gauting cohort

In the following, we report the data of the comprehensive data set from the Hamburg/Gauting cohort, consisting of 490 patients from seven centers.

Baseline characteristics and baseline symptoms of this cohort are shown in Table 4.

**Table 4:** Baseline characteristics and baseline symptoms of cohort Hamburg/Gauting (n = 490).

	N	(N = 490)
**Outcomes**		
Admission to intensive care unit (n)	490	0.26 (126)
Death (n)	490	0.2 (97)
Ventilation (n)	490	0.17 (83)
**Baseline Symptoms**		
Presence of cough (n)	453	0.58 (264)
Presence of fever (>38·5°C) (n)	448	0.46 (205)
Presence of dyspnea (n)	448	0.57 (256)
**Baseline vital parameters**		
Heart rate, /min	451	76/ 86/100 88+/- 19
Systolic blood pressure, mmHg	446	120/130/145 133+/- 22
Respiratory rate, /min	356	15/18/22 20+/- 6
spO2, %	442	91.0/95.0/97.0 93.3+/- 5.8
**Baseline laboratory findings**		
Lymphocytes, /nl	294	0.66/1.00/1.40 1.73+/-3.48
Leukocytes, /nl	478	5.1/6.9/9.6 7.9+/-4.1
Neutrophile, /nl	315	3.3/ 4.8/ 7.3 6.5+/-10.9
D-Dimer, mg/l	217	0.53/ 1.02/ 2.12 4.41+/-14.51
Creatinine, mg/dl	474	0.8/1.0/1.4 1.4+/-1.6
CRP, mg/l	474	23/ 64/118 84+/- 80
LDH, U/I	358	252/322/448 417+/-745
PTT, seconds	370	29/32/36 35+/-16
Potassium, mmol/l	455	3.7/4.0/4.4 4.0+/-0.6
Lactate, mmol/dl	347	0.9/ 1.3/ 1.9 2.9+/-14.5
Number of comorbidities*:		
0	445	0.27 (122)
1		0.22 (100)
2		0.21 (95)
3		0.15 (67)
4		0.09 (39)
5		0.03 (15)
6		0.02 (7)
Tumor disease (n)	490	0.19 (93)
Imaging performed (n)	490	0.81 (398)
**Medication**		
Antiplatelet medication (n)	490	0.27 (130)
ACE-Inhibitor/Angiotensin-receptor-blocker (n)	490	0.42 (204)
Antidiabetic medication (oral and insulin, insulinanaloga) (n)	490	0.22 (106)
Immuno-suppressive medication (n)	490	0.18 (87)
Statins (n)	459	0.21 (96)
Oral anticoagulation (n)	467	0.14 (67)
Diuretics (n)	457	0.24 (110)
Prednisolone (n)	454	0.04 (20)
Proton pump inhibitor (n)	453	0.3 (135)

N are number of non-missing values. Continuous data are shown as mean ± standard deviation, first quartile/median/third quartile. Categorical data as proportions (n).

*Comorbidities include presence of cardiomyopathy, hypertension, diabetes mellitus, vascular disease, dyslipidemia, any prior pulmonary disease, chronic kidney disease, any active tumor disease.

### Primary endpoint

Of the included 490 patients, 63% were classified as mild disease, 17% as severe (ICU/ventilation, non-fatal), and 20% as fatal (death), respectively (Fig 2). For the analysis as potential risk factor 25 of 45 variables remained after excluding values with systematic missing or irrelevant data.

**Fatal disease (mortality)** in all Hamburg/Gauting patients was predicted by age (odds ratio (OR) 10.712, 95-%-confidence interval (CI): [3.773–30.412], baseline leukocytes (OR 1.367, CI: [1.016–1.839], CRP (OR 2.277 [1.637–3.166]), creatinine (OR 2.306, CI: [1.275–4.172], and use of oral anticoagulation (OR 1.676, CI: [0.875–3.210]).

**Fig 6:**
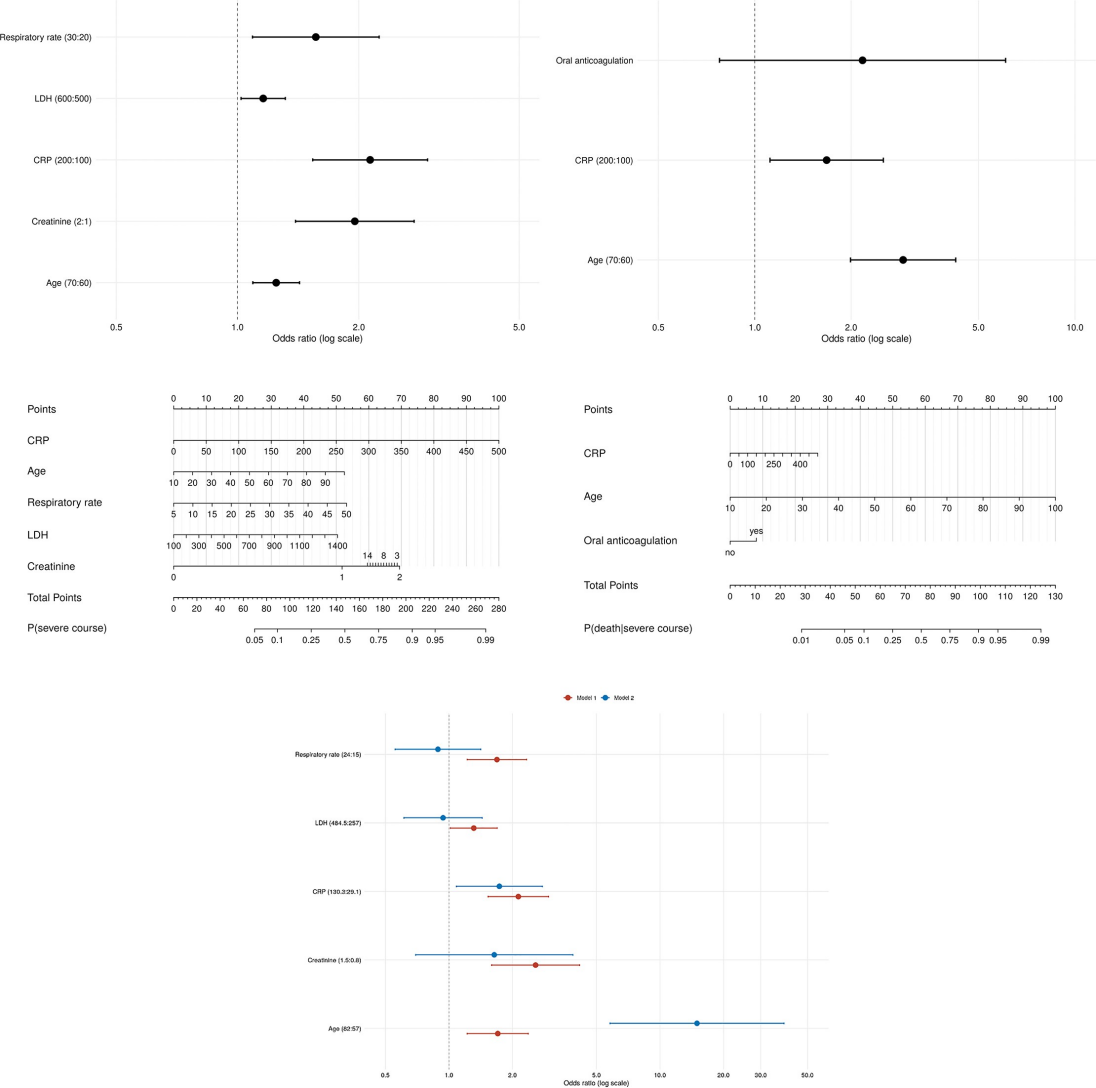
Predicting severity of disease from baseline data: Fig 6A (above) shows the interquartile-range odds ratios for continuous predictors (upper quartile: lower quartile) and simple odds ratios for categorical predictors (current category: reference category) with regard to a severe or fatal disease. **Fig 6A (below)** shows the nomogram (risk score): calculating the probability for a severe or fatal disease. For each predictor, points (0–100) are assigned. The total points are associated to event prediction. E.g. In case of an 80 years old (= 40 points) patient with CRP 150 (= 30 points), respiratory rate 24 (= 30 points), LDH 400 (= 10 points), and creatinine 2 (= 73 points), the total point score is 183 and therefore the risk for ventilation, ICU, or death is elevated with a probability of approximately 0.7. **Fig 6B (above)** shows the interquartile-range odds ratios for continuous predictors (upper quartile: lower quartile) and simple odds ratios for categorical predictors (current category: reference category) with regard to a fatal disease conditional of patients with at least severe diseases (mild courses are not taken into account). **Fig 6B (below)** shows the nomogram (risk score): calculating the probability for a fatal course of patients with severe diseases. **Fig 6C** This figure shows comparing effects of the above mentioned two models (model 1 for prediction of the combined endpoint versus model 2 for prediction of death in mechanical ventilated/ICU patients) by baseline parameters. The interquartile-range odds ratios for continuous predictors (upper quartile: lower quartile) and simple odds ratios for categorical predictors (current category: reference category) with regard to disease severity (Model 1) and mortality (Model 2) are compared.

**Occurrence of severe and fatal disease** (model 1) was associated with age (OR 1.704, CI: [1.221–2.377]), baseline respiratory rate (OR 1.688, CI: [1.222–2.333]), lactate dehydrogenase (LDH), (OR 1.312, CI: [1.015–1.695]), C-reactive protein (CRP) (OR 2.132, CI: [1.533–2.965]), and creatinine values (2.573, CI: [1.593–4.154]). A nomogram (risk score) derived from the regression model was designed to predict the probability of a severe disease from baseline data (Fig 6A).

**For fatal outcome in patients with a severe disease** (at least mechanical ventilation and/or ICU admission during hospitalization, model 2) age (OR 14.322, CI: [5.565–36.862]), baseline CRP (OR 1.699, CI: [1.123–2.571]), and the baseline use of oral anticoagulation (OR 2.183, CI: [0.780–6.109]) were independent risk factors. The corresponding nomogram (risk score) is shown in Fig 6B.

When comparing common effects in both models the role of age largely increased, whilst other effects (respiratory rate, LDH, CRP, and creatinine) were negligibly smaller (Fig 6C).

## Discussion

Major findings of the study showed that more than one third of all hospitalized COVID-19 patients suffered from a fatal or severe disease requiring mechanical ventilation and or admission to intensive care unit. The risk for a severe and/or fatal disease can be calculated by a simple risk score, since age, respiratory rate, lactate dehydrogenase, C-reactive protein, and creatinine at baseline are associated with this outcome. Additionally, mortality rates of hospitalized COVID-19 patients showed temporal variations with lower rates during summer. Age had a big effect on in-hospital mortality in severe diseased patients and also had a strong influence on the temporal changes of mortality rates. SARS-CoV-2 variants of concern did not play a role in this study, as in Germany the first detected case was at the end of December 2020 [3].

### Risk factors and risk score

The identified risk factors for all cause death and need for ventilation or ICU treatment in our study are in line with the results of Galloway et al. who also identified respiratory rate, higher CRP, and renal impairment as relevant risk factors among others; however, their study approach was slightly different [9]. The overall strongest predictor in our study was age, especially in ICU-patients. The importance of age in COVID-19 severity was reported previously and confirmed by national health monitoring in Germany, as 85% of all fatal cases in Germany occurred in patients ≥70 years of age [3].

Interestingly, the examined coexisting comorbidities did not show a strong effect in our study, which should not mistakenly lead to the impression that our findings are controversial to other studies that clearly showed comorbidities to be a predictor for mortality [14]. Our analysis only demonstrates them to be weaker than other factors. Only the pre-existing use of oral anticoagulation was a strong predictor for severe outcome and mortality, which might point to atrial fibrillation as a relevant risk factor.

To further optimize the detection of high-risk COVID-19 patients, we developed a simple risk score (nomogram), which can be used in daily routine. Baseline characteristics such as age, laboratory values, and respiratory rate give a specific point value for each patient adding up to the individual patient’s risk. This risk score is based on a comprehensive and highly validated, prospective dataset with an integrated approach of data analysis and is therefore of high quality. Hence, we overcome some limitations of other published risk scores with retrospective analysis or smaller sample sizes [15]. In contrast to the approach of Nakakubo et al. who included diagnostic imaging to the risk score [16], we focused on simple baseline characteristics and laboratory values, accessible for general practitioners or clinicians in emergency departments. Especially in the current pandemic situation with further rising infection rates worldwide, it is necessary to detect vulnerable patients fast and easily.

### Temporal variations

The COVID-19 disease causes increased morbidity and mortality around the globe. The “CORONA Germany” study was carried out by one of the largest hospital networks in Germany with clinics in nearly all states of the country (as previously shown in Fig 1). During the observational period, we enrolled a large number of COVID-19 patients in 45 hospitals with a mortality rate as high as 19%. Whereas in-hospital mortality in Britain was described as 37% [17], analysis for Germany showed rates ranging from 17% to 22.2% [3, 8].

Since the beginning of the pandemic in Germany, SARS-CoV-2 infection rates and numbers of hospitalized COVID-19 patients emerged in different phases, as shown in our study with a first peak in April 2020 and comparatively low hospital admission rates during summer. The onset of a second wave was observed in September 2020 with still rising numbers of COVID-19 hospitalized patients in December 2020 [3]. Interestingly, mortality rates of hospitalized patients in this study also showed temporal variations similar to the seasonal peaks of admission numbers. Whereas in-hospital mortality in April and December 2020 was 22.4% and 23.1%, it dropped to 3% in June, even though all patients had a clinical indication for hospitalization. Furthermore, rates of pneumonia, respiratory insufficiency, and the need for mechanical ventilation showed similar trends, which may indicate to a higher proportion of less severe courses of the disease during summer season. Dennis et al. previously described a decrease of in-hospital mortality in a big cohort of intensive care patients from March until June 2020, which was assumed to be due to introduction of effective treatments and a learning curve regarding clinical management of the novel disease [6]. However, this would not explain the increase of in-hospital mortality from August to December observed in our study.

Regression analyses in our study demonstrate the observed temporal variation in mortality to be strongly influenced by age. In addition to this strong association, other factors might also play a role and we assume seasonality of COVID-19 to be noteworthy. Global SARS-Cov-2 infection rates were presumed to underlie seasonality [5]. Among other factors, this was explained by the fact that UV light is most strongly associated with lower COVID-19 growth [18]. Additionally, potential co-infectious diseases also consist of seasonal variations like e.g. pneumococcal infection [19]. Because the prevalence of co-infection was reported to be up to 50% among non-survivors in COVID-19 [20], this may be an additional factor explaining the seasonal changes of in-hospital mortality over the year. Of course, due to the novelty of SARS-CoV-2, it is too early to prove seasonality; nevertheless, it may be an additional cause for the temporal pattern of in-hospital mortality beside the strong association to age.

### Limitations

As we present all patients being hospitalized and tested positive for SARS-CoV2, the study population is heterogeneous and no further inclusion criteria were applied. Patients were at different states of COVID-19 disease by the time of admission and this could bias our results. However, some of our findings have been reported in comparable studies. Additionally, the effect of specific treatments has not been analyzed in this study and therefore it is unclear whether specific treatment options had an influence on the patients’ outcome. However, as treatment options for COVID-19 are still the subject of current research, their impact is still mostly unknown. Despite high quality data collection and endpoint review committee, all data are observational and taken of medical patient charts during hospitalization. This has led to missing values in the study cohort. Therefore, their role might be underestimated in predicting the study outcome.

## Conclusions

In conclusion, risk factors such as age, respiratory rate, lactate dehydrogenase, C-reactive protein, and creatinine values at baseline are associated with all cause death, need for ventilation, and/or ICU treatment in a nationwide series of COVID 19 hospitalized patients. Especially age plays an important prognostic role for mortality in critically ill patients. A simple risk score was developed to assist general practitioners or clinicians in emergency departments to detect vulnerable patients fast and easily.

Additionally, we observed temporal variations of in-hospital mortality and morbidity during the year 2020. In-hospital mortality of COVID-19 raised again by the end of the year, after a prior dip during the summer months. Again, the temporal course of mortality was influenced by age, highlighting the importance of this consistent risk factor.

## Supporting information

S1 Appendix List of participating hospitals. (PDF)

S2 Appendix Validation results. (PDF)

S3 Appendix References regarding the statistical analysis. (PDF)
