# Supplementary material for: Clinical outcome, risk assessment, and seasonal variation in hospitalized COVID-19 patients—Results from the CORONA Germany study
Source: PLoS One. 2021 Jun 17;16(6):e0252867. doi: 10.1371/journal.pone.0252867 (PMC8211271; doi:10.1371/journal.pone.0252867)
Supplement: S1 Appendix — (PDF) [file pone.0252867.s001.pdf]

S1 Appendix. List of participating hospitals

	Hospital name	City	Region in Germany
1.	Asklepios Klinik Altona	Hamburg	North
2.	Asklepios Klinikum Bad Abbach	Bad Abbach	South
3.	Asklepios Schlossberg Klinik Bad König	Bad König	South-West
4.	Asklepios Klinik Bad Oldesloe	Bad Oldesloe	North
5.	Asklepios Neurologische Klinik Bad Salzhausen	Bad Salzhausen	South-West
6.	Asklepios Stadtklinik Bad Tölz	Bad Tölz	South
7.	Asklepios Klink Fürstenhof Bad Wildungen	Bad Wildungen	South-West
8.	Asklepios Stadtklinik Bad Wildungen	Bad Wildungen	South-West
9.	Asklepios Klinik Barmbek	Hamburg	North
10.	Asklepios Klinik Birkenwerder	Birkenwerder	North-East
11.	Asklepios Fachklinikum Brandenburg	Brandenburg an der Havel	North-East
12.	Asklepios Klinik im Städtedreieck	Burglengenfeld	South
13.	Asklepios Neurologische Klinik Falkenstein	Königstein-Falkenstein	South-West
14.	Asklepios Fachkliniken München-Gauting	Gauting	South
15.	Asklepios Harzklinik Goslar	Goslar	North-West
16.	Asklepios Klinikum Harburg	Hamburg	North
17.	Asklepios Orthopädische Klinik Hohwald	Neustadt in Sachsen	South-East
18.	Asklepios Klinik Langen	Langen	South-West
19.	Asklepios Klinik Lich	Lich	South-West
20.	Asklepios Privatklinik Lich	Lich	South-West
21.	Asklepios Klinik Lindau	Lindau	South
22.	Asklepios Orthopädische Klinik Lindenlohe	Schwandorf	South
23.	Asklepios Fachklinikum Lübben	Lübben	North-East
24.	Asklepios Klinikum Melsungen	Melsungen	South-West
25.	Asklepios Klinik Nord Heidberg	Hamburg	North
26.	Asklepios Klinik Oberviechtach	Oberviechtach	South
27.	Asklepios Klinik Parchim	Parchim	North-East
28.	Asklepios Klinik Pasewalk	Pasewalk	North-East
29.	Asklepios-ASB Krankenhaus Radeberg	Radeberg	South-East
30.	Asklepios Klinik Sankt Augustin	Sankt Augustin	North-West
31.	Asklepios Klinikum Schwalmstadt	Schwalmstadt	South-West
32.	Asklepios Klinikum Uckermark	Schwedt/ Oder	North-East
33.	Sächsische Schweiz Kliniken	Sebnitz	South-East
34.	Asklepios Akutklinik Seesen	Seesen	North-West
35.	Asklepios Klinik Seligenstadt	Seligenstadt	South-West
36.	Asklepios Klinik St. Georg	Hamburg	North
37.	Asklepios Fachklinikum Stadtroda	Stadtroda	South-East
38.	Asklepios Südpfalzklinik Germersheim	Germersheim	South-West
39.	Asklepios Fachklinikum Teupitz	Teupitz	North-East
40.	Asklepios Klinik Wandsbek	Hamburg	North
41.	Asklepios Klinik Weißenfels	Weißenfels	South-East
42.	Asklepios Nordseeklinik Westerland	Sylt - OT Westerland	North
43.	Asklepios Westklinikum Rissen	Hamburg	North
44.	Asklepios Paulinen Klinik Wiesbaden	Wiesbaden	South-West
45.	Asklepios Privatklinik Wiesbaden	Wiesbaden	South-West
