# Supplementary material for: Clinical outcome, risk assessment, and seasonal variation in hospitalized COVID-19 patients—Results from the CORONA Germany study
Source: PLoS One. 2021 Jun 17;16(6):e0252867. doi: 10.1371/journal.pone.0252867 (PMC8211271; doi:10.1371/journal.pone.0252867)
Supplement: S2 Appendix — (PDF) [file pone.0252867.s002.pdf]

S2 Appendix. Validation results

Model: severe disease

Table OR

term	estimate	conf.low	conf.high
CRP (200:100)	2.1	1.5	3.0
Age (70:60)	1.2	1.1	1.4
Respiratory rate (30:20)	1.6	1.1	2.2
LDH (600:500)	1.2	1.0	1.3
Creatinine (2:1)	2.0	1.4	2.7

OR Plot

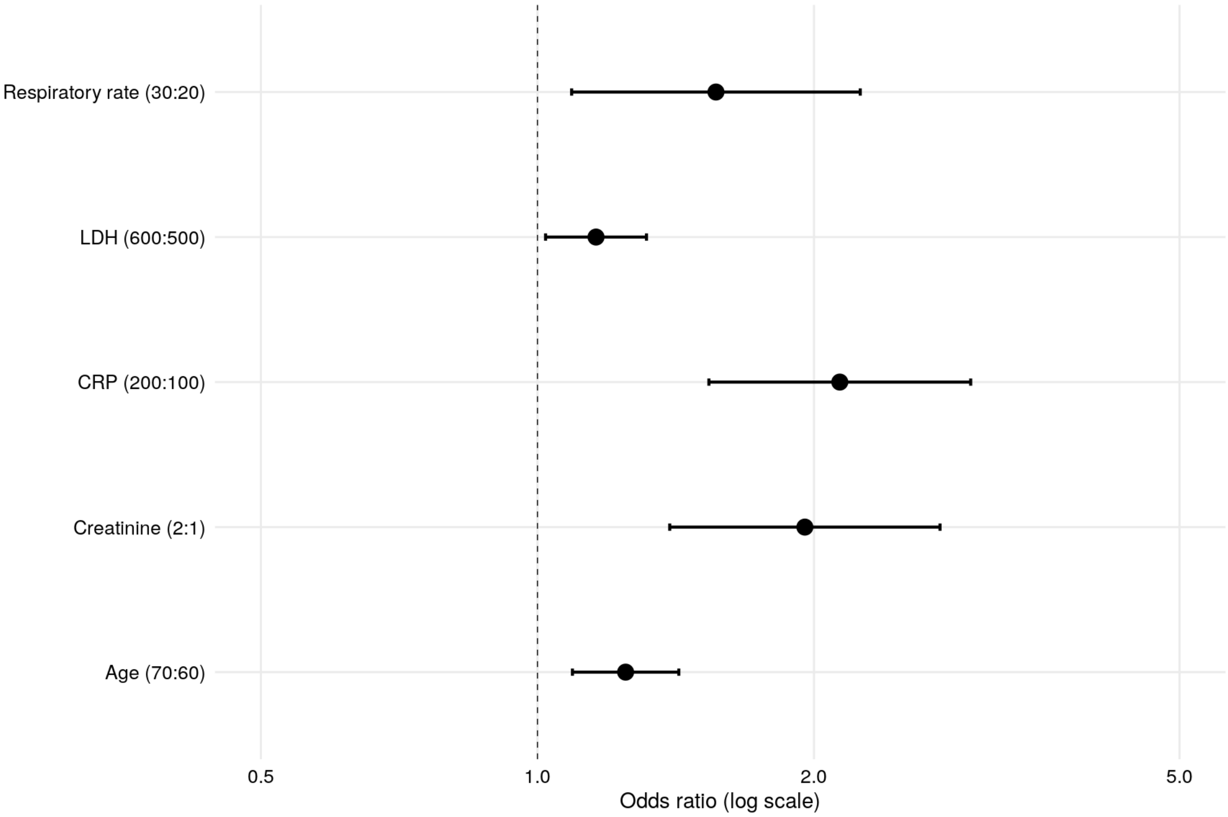

Validation

term	index.orig	training	test	optimism	index.corrected	n
Dxy	0.544	0.550	0.535	0.015	0.529	40
R2	0.292	0.296	0.281	0.016	0.276	40
Intercept	0.000	0.000	-0.013	0.013	-0.013	40
Slope	1.000	1.000	0.953	0.047	0.953	40
Emax	0.000	0.000	0.013	0.013	0.013	40
D	0.238	0.242	0.228	0.014	0.224	40
U	-0.004	-0.004	0.001	-0.005	0.001	40
Q	0.242	0.246	0.227	0.019	0.224	40
B	0.179	0.176	0.182	-0.005	0.185	40
g	1.353	1.376	1.313	0.063	1.289	40
gp	0.257	0.256	0.251	0.004	0.252	40

Calibration Plot

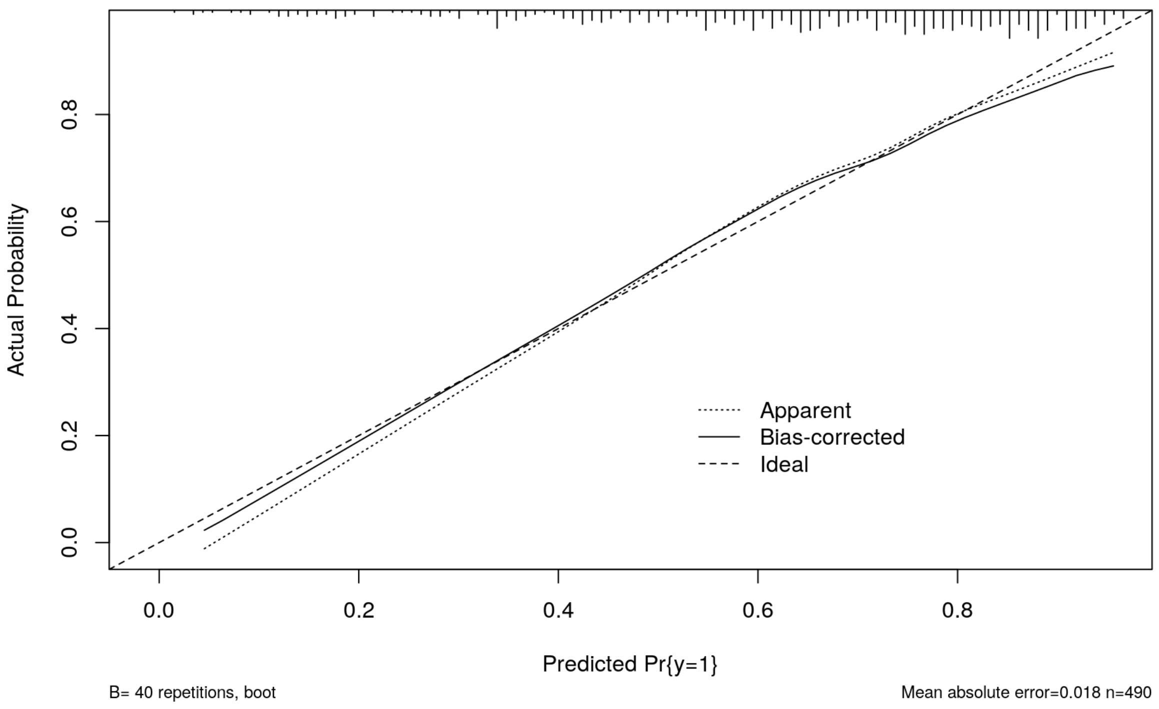

Model: death conditional on severe disease

Table OR

term	estimate	conf.low	conf.high
CRP (200:100)	1.7	1.12	2.5
Age (70:60)	2.9	1.99	4.2
Oral anticoagulation	2.2	0.78	6.1

OR Plot

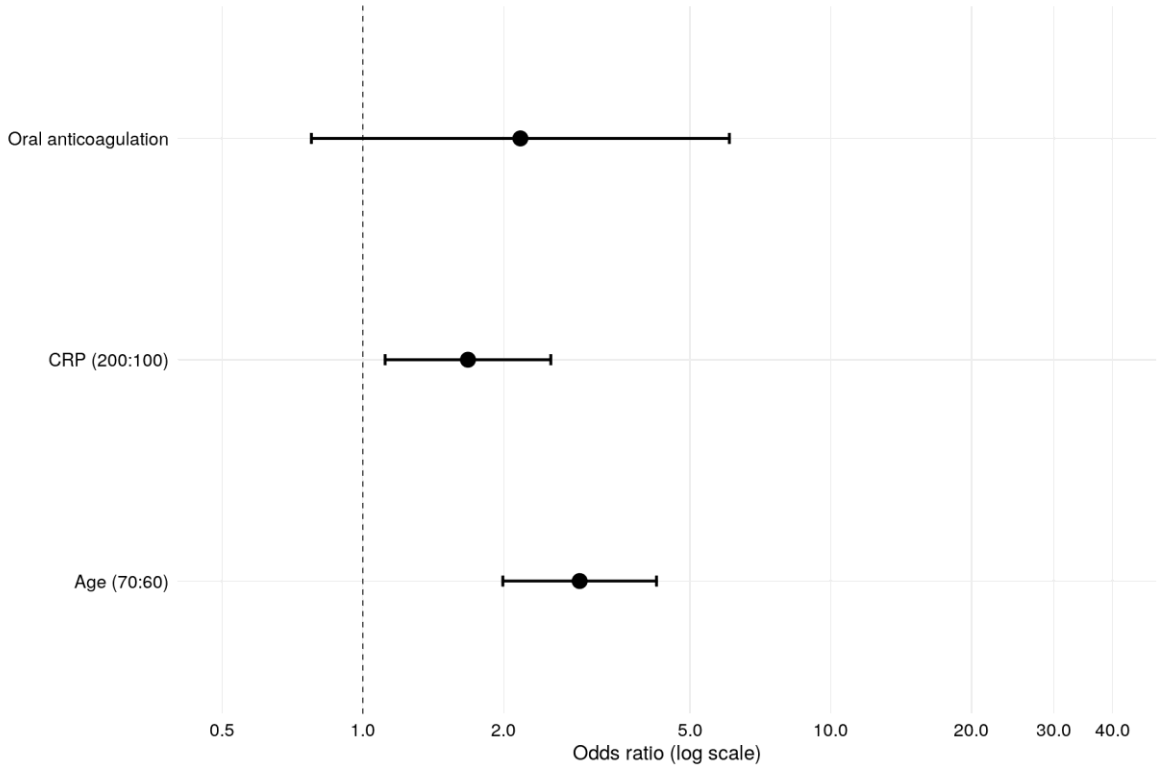

Validation

term	index.orig	training	test	optimism	index.corrected	n
Dxy	0.687	0.700	0.668	0.032	0.655	40
R2	0.443	0.475	0.432	0.044	0.400	40
Intercept	0.000	0.000	0.009	-0.009	0.009	40
Slope	1.000	1.000	0.904	0.096	0.904	40
Emax	0.000	0.000	0.024	0.024	0.024	40
D	0.398	0.435	0.385	0.050	0.348	40
U	-0.011	-0.011	0.005	-0.016	0.005	40
Q	0.409	0.446	0.380	0.066	0.343	40
B	0.164	0.155	0.168	-0.013	0.177	40
g	2.039	2.250	1.981	0.269	1.770	40
gp	0.335	0.345	0.330	0.015	0.320	40

Calibration Plot

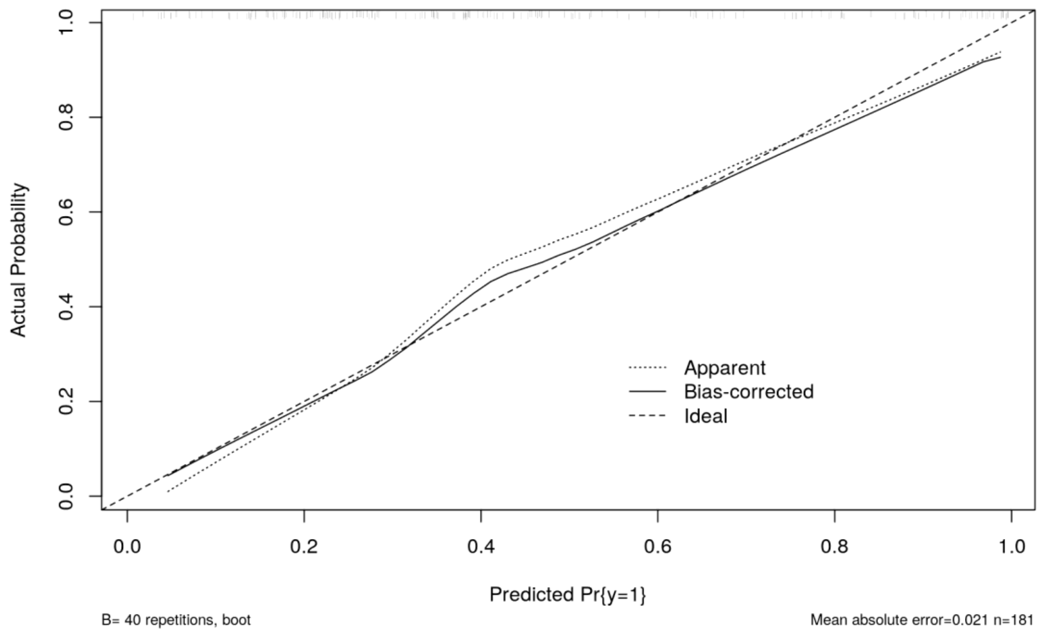

Validation explanation

Bias-corrected Somers' D_{xy} rank correlation, R-squared index, the intercept and slope of an overall logistic calibration equation, the maximum absolute difference in predicted and calibrated probabilities E_{max}, the discrimination index D (model L.R. (chi-square - 1)/n), the unreliability index U = difference in -2 log likelihood between un-calibrated X beta and X beta with overall intercept and slope calibrated to test sample / n, the overall quality index (logarithmic probability score) Q = D - U, and the Brier or quadratic probability score, B (the last 3 are not computed for ordinal models), the g-index, and gp, the g-index on the probability scale.
