# Supplementary material for: Clinical outcome, risk assessment, and seasonal variation in hospitalized COVID-19 patients—Results from the CORONA Germany study
Source: PLoS One. 2021 Jun 17;16(6):e0252867. doi: 10.1371/journal.pone.0252867 (PMC8211271; doi:10.1371/journal.pone.0252867)
Supplement: S3 Appendix — (PDF) [file pone.0252867.s003.pdf]

S3 Appendix. References regarding the statistical analysis

1. Allaire, JJ, Yihui Xie, Jonathan McPherson, Javier Luraschi, Kevin Ushey, Aron Atkins, Hadley Wickham, Joe Cheng, Winston Chang, and Richard Iannone. 2020. Rmarkdown: Dynamic Documents for R. https://github.com/rstudio/rmarkdown.

2. Francois, Romain. 2017. *Bibtex: Bibtex Parser*. https://CRAN.R-project.org/package=bibtex.

3. Harrell Jr, Frank E, with contributions from Charles Dupont, and many others. 2020. *Hmisc: Harrell Miscellaneous*. https://CRAN.R-project.org/package=Hmisc.

4. R Core Team. 2020. *R: A Language and Environment for Statistical Computing*. Vienna, Austria: R Foundation for Statistical Computing. https://www.R-project.org/.

5. Xie, Yihui, J. J. Allaire, and Garrett Grolemund. 2018. *R Markdown: The Definitive Guide*. Boca Raton, Florida: Chapman; Hall/CRC. https://bookdown.org/yihui/rmarkdown.

6. Xie, Yihui, Christophe Dervieux, and Emily Riederer. 2020. R Markdown Cookbook. Boca Raton, Florida: Chapman; Hall/CRC. https://bookdown.org/yihui/rmarkdown-cookbook.
